# Synthesis and Evaluation of ^99m^Tc-Labeled PSMA-Targeted Tracers Based on the Lys-Urea-Aad Pharmacophore for Detecting Prostate Cancer with Single Photon Emission Computed Tomography

**DOI:** 10.3390/molecules28135120

**Published:** 2023-06-29

**Authors:** Kelly Lu, Chengcheng Zhang, Zhengxing Zhang, Hsiou-Ting Kuo, Nadine Colpo, François Bénard, Kuo-Shyan Lin

**Affiliations:** 1 Department of Molecular Oncology, BC Cancer Research Institute, Vancouver, BC V5Z 1L3, Canada; klu@bccrc.ca (K.L.); cczhang@bccrc.ca (C.Z.); zx1981ok@hotmail.com (Z.Z.); hkuo@bccrc.ca (H.-T.K.); ncolpo@bccrc.ca (N.C.); fbenard@bccrc.ca (F.B.); 2 Department of Functional Imaging, BC Cancer Research Institute, Vancouver, BC V5Z 4E6, Canada; 3 Department of Radiology, University of British Columbia, Vancouver, BC V5Z 1M9, Canada

**Keywords:** prostate-specific membrane antigen (PSMA), technetium-99m, single photon emission computed tomography (SPECT), molecular imaging, HYNIC

## Abstract

Prostate-specific membrane antigen (PSMA) is a well-validated prostate cancer marker but reported PSMA-targeted tracers derived from the Lys-urea-Glu pharmacophore including the clinically validated [^99m^Tc]Tc-EDDA/HYNIC-iPSMA have high off-target uptake in kidneys, spleen, and salivary glands. In this study, we synthesized and evaluated three novel ^99m^Tc-labeled PSMA-targeted tracers and investigated if the tracers derived from the Lys-urea-Aad pharmacophore could have minimized uptake in off-target organs/tissues. In vitro competition binding assays showed that compared with HYNIC-iPSMA, the three novel ligands had slightly weaker PSMA binding affinity (average K_i_ = 3.11 vs. 8.96–11.6 nM). Imaging and ex vivo biodistribution studies in LNCaP tumor-bearing mice showed that [^99m^Tc]Tc-EDDA/HYNIC-iPSMA and the three novel tracers successfully visualized LNCaP tumor xenografts in SPECT images and were excreted mainly via the renal pathway. The average tumor uptake at 1 h post-injection varied from 5.40 to 18.8%ID/g, and the tracers derived from the Lys-urea-Aad pharmacophore had much lower uptake in the spleen and salivary glands. Compared with the clinical tracer [^99m^Tc]Tc-EDDA/HYNIC-iPSMA, the Lys-urea-Aad-derived [^99m^Tc]Tc-EDDA-KL01127 had lower background uptake and superior tumor-to-background contrast ratios and is therefore promising for clinical translation to detect prostate cancer lesions with SPECT.

## 1. Introduction

Prostate cancer (PCa) is one of the leading causes of cancer mortality in men worldwide. Currently, its incidence and mortality have plateaued potentially due to limitations of current diagnostic and therapeutic options for PCa [1]. Hence, more effective molecular and functional-based imaging using radiotracers targeting PCa markers may provide enhanced diagnosis and monitoring of the disease. Prostate-specific membrane antigen (PSMA), also known as glutamate carboxypeptidase II (GCPII), is a biomarker expressed on epithelial prostate cells but found to be overexpressed in PCa and its expression correlates with disease progression, recurrence, metastasis, and survival even in patients with castration-resistance PCa [2,3,4].

**Figure 1:**
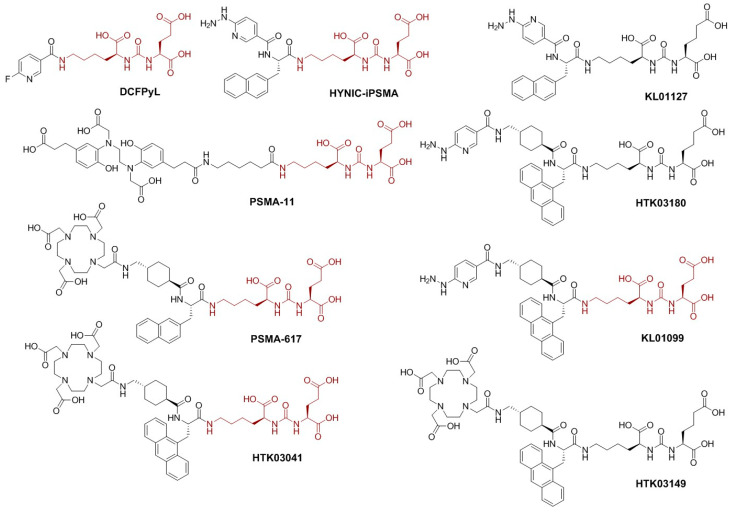
Chemical structures of PSMA-targeted ligands. The Lys-urea-Glu pharmacophore is brown.

PSMA features an extracellular active site which catalyzes the hydrolysis of *N*-acetyl-aspartyl-glutamate into *N*-acetyl-aspartate and glutamate and is also the binding site of most currently-reported PSMA-targeted small molecules [5,6]. Among PSMA inhibitors, urea-based small molecules provide stability and an opportunity for cell internalization and have been extensively explored as PSMA-targeted tracers for positron emission tomography (PET) imaging such as [^18^F]DCFPyL and [^68^Ga]Ga-PSMA-11 (Figure 1) to single photon emission computed tomography (SPECT) imaging such as [^99m^Tc]Tc-EDDA/HYNIC-iPSMA (Figure 1). These reported PSMA-targeted tracers all share the same lysine-urea-glutamate (Lys-urea-Glu, in brown, Figure 1) pharmacophore [7,8]. However, a limitation to utilizing the Lys-urea-Glu pharmacophore is the off-target accumulation in some normal organs/tissues, including kidneys, spleen, salivary glands, and liver, which could deter detection of metastatic PCa lesions in or adjacent to these organs/tissues [9,10]. Our previous work demonstrated that a PSMA-targeted PET tracer, [^68^Ga]Ga-HTK03149 (Figure 1), derived from a modified lysine-urea-aminoadipic acid (Lys-urea-Aad) pharmacophore had minimal uptake in the commonly reported off-target organs including kidneys, spleen, salivary glands, and liver [11]. Hence, the goal of this study is to investigate if the same effect could be observed in the development of ^99m^Tc-labeled tracers for SPECT imaging.

Despite the higher sensitivity and spatial resolution of PET, SPECT is more readily available and accessible in hospital settings. Therefore, an optimized PSMA-targeted SPECT tracer labeled with ^99m^Tc could have wider applicability than FDA-approved PET tracers, [^18^F]DCFPyL and [^68^Ga]Ga-PSMA-11. Moreover, ^99m^Tc-based radiopharmaceuticals are easy to prepare from kit formulations and feature a longer half-life (6 h) for efficient utilization.

The clinically validated [^99m^Tc]Tc-EDDA/HYNIC-iPSMA features a hydrazinonicotinamide (HYNIC) chelator which utilizes the co-ligand, ethylenediamine-*N*,*N’*-diacetic acid (EDDA) to complete the coordination of technetium under relatively mild conditions that can be facilely developed into freeze-dried kit formulation for clinical use [12,13,14]. Despite its capability to detect PSMA-expressing PCa lesions, high uptake of [^99m^Tc]Tc-EDDA/HYNIC-iPSMA was also observed in the reported off-targets of other PSMA-targeted tracers, including salivary glands, liver, kidneys, and spleen. Therefore, in this study, we used [^99m^Tc]Tc-EDDA/HYNIC-iPSMA as a template and investigated if (1) replacing the PSMA-targeted Lys-urea-Glu pharmacophore with the Lys-urea-Aad pharmacophore could lead to new derivatives with lower off-target uptake in normal organs, and (2) optimizing the selection of linker between the PSMA-targeted pharmacophore and the HYNIC chelator could lead to new derivatives with higher PSMA binding affinity and/or higher uptake in PSMA-expressing PCa cancer tumor xenografts.

As shown in Figure 1, we synthesized HTK03180, KL01099, and KL01127 and compared the performance of their ^99m^Tc-labeled complexes with [^99m^Tc]Tc-EDDA/HYNIC-iPSMA. HYNIC-iPSMA is a derivative of PSMA-617 (Figure 1) by replacing the DOTA-tranexamic acid moiety with the HYNIC chelator. Previously, we demonstrated that tranexamic acid is crucial for high binding affinity to PSMA [15]. Therefore, we preserved tranexamic acid as part of the lipophilic linker for HTK03180 and KL01099. Previously, we also demonstrated that replacing the 2-naphthylalanine in [^68^Ga]Ga-PSMA-617 with 9-anthrylalaine resulted in [^68^Ga]Ga-HTK03041 (Figure 1), which had higher PSMA binding affinity and uptake in PSMA-expressing LNCaP tumor xenografts than [^68^Ga]Ga-PSMA-617 [16]. We would like to investigate if the same effect could be observed in ^99m^Tc-labeled HYNIC-conjugated tracers (HTK03180 and KL01099, Figure 1). Lastly, instead of the traditional PSMA-targeted Lys-urea-Glu pharmacophore in HYNIC-iPSMA and KL01099, we used the Lys-urea-Aad pharmacophore in HTK03180 and KL01127. This was to investigate if the ^99m^Tc-labeled tracer derived from the Lys-urea-Aad pharmacophore could have lower off-target uptake in normal organs than its Lys-urea-Glu analog (HTK03180 vs. KL01099 and HYNIC-iPSMA vs. KL01127, Figure 1).

## 2. Results

### 2.1. Synthesis of PSMA-Targeted Ligands and Their Tc-99m Labeled Analogs

PSMA-targeted ligands, including HYNIC-iPSMA, HTK03180, KL01099, and KL01127, were constructed in solid phase and obtained an 18–36% yield after HPLC purification. Their Tc-99m labeled analogs were obtained in 63–89% decay-corrected radiochemical yield with >97% radiochemical purity and >55 GBq/µmol molar activity (Figures S1–S4).

### 2.2. PSMA Binding Affinity and Hydrophilicity

**Figure 2:**
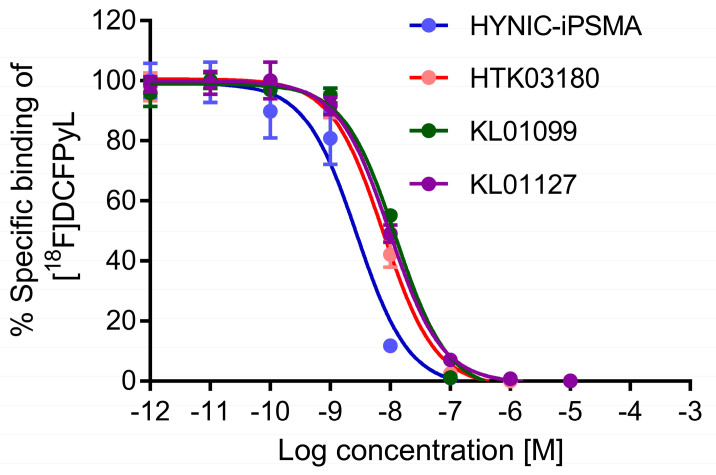
Inhibition of [^18^F]DCFPyL binding to PSMA-expressing LNCaP cells by various concentrations of HYNIC-iPSMA, HTK03180, KL01099, and KL01127.

All tested PSMA-targeted ligands inhibited the binding of [^18^F]DCFPyL to PSMA-expressing LNCaP cells in a concentration-dependent manner (Figure 2, *n* = 3). Their calculated K_i_ values were in the nM range, ranking from the highest binding with HYNIC-iPSMA (3.11 ± 0.76 nM), followed by KL01127 (8.96 ± 0.58 nM), KL01099 (10.71 ± 0.21 nM), and HTK03180 (11.6 ± 1.5 nM). HYNIC-iPSMA had a significantly higher binding affinity than the other three ligands (ANOVA, *p* < 0.001), whereas there was no significant difference between the binding affinities of KL01127, KL01099, and HTK03180 (ANOVA, *p* > 0.05).

The hydrophilicity of the ^99m^Tc-labeled tracers was measured via the shake-flask method with the LogD_7.4_ values calculated to be −3.31 ± 0.02, −2.56 ± 0.02, −2.14 ± 0.01, and −3.37 ± 0.11 (*n* = 3) for [^99m^Tc]Tc-EDDA/HYNIC-iPSMA, [^99m^Tc]Tc-EDDA-HTK03180, [^99m^Tc]Tc-EDDA-KL01099, and [^99m^Tc]Tc-EDDA-KL01127, respectively. Analyses by *t*-test revealed that [^99m^Tc]Tc-EDDA-HTK03180 is more hydrophilic than [^99m^Tc]Tc-EDDA-KL01099 (−2.56 ± 0.02 vs. −2.14 ± 0.01; *p* < 0.01), whereas no difference was observed between [^99m^Tc]Tc-EDDA/HYNIC-iPSMA and [^99m^Tc]Tc-EDDA-KL01127 (−3.31 ± 0.02 vs. −3.37 ± 0.11; *p* > 0.1).

### 2.3. Ex Vivo Biodistribution and SPECT/CT Imaging Studies

**Figure 3:**
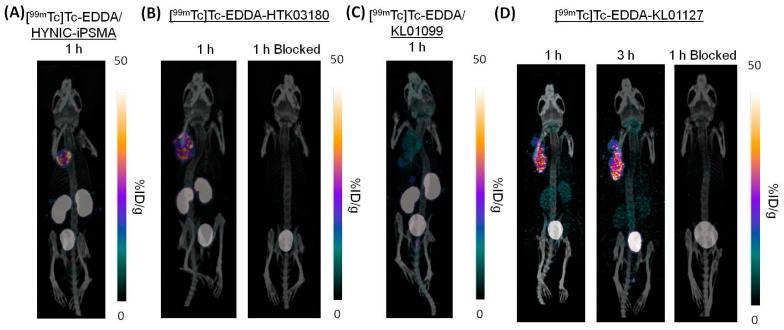
Representative SPECT/CT images with LNCaP tumor in the left shoulder of (**A**) [^99m^Tc]EDDA/HYNIC-iPSMA, (**B**) [^99m^Tc]Tc-EDDA-HTK03180, (**C**) [^99m^Tc]Tc-EDDA-KL01099, and (**D**) [^99m^Tc]Tc-EDDA-KL01127 in LNCaP tumor-bearing mice. The mice in the blocked groups were co-injected with 0.5 mg of 2-PMPA.

Representative SPECT/CT images of the investigated ^99m^Tc-labeled PSMA-targeted tracers in LNCaP tumor-bearing mice are shown in Figure 3. All tracers successfully visualized LNCaP tumor xenografts in SPECT images with good contrast and were excreted mainly via the renal pathway. [^99m^Tc]Tc-EDDA/HYNIC-iPSMA (Figure 3A), [^99m^Tc]Tc-EDDA-HTK03180 (Figure 3B), and [^99m^Tc]Tc-EDDA-KL01127 (Figure 3D) had high tumor uptake, and [^99m^Tc]Tc-EDDA-KL01099 (Figure 3C) had relatively lower tumor uptake. [^99m^Tc]Tc-EDDA/HYNIC-iPSMA, [^99m^Tc]Tc-EDDA-HTK03180, and [^99m^Tc]Tc-EDDA-KL01099 had very high uptake in kidneys, but only moderate kidney uptake was observed using [^99m^Tc]Tc-EDDA-KL01127. The tumor uptake of [^99m^Tc]Tc-EDDA-KL01127 was sustained over time, while its kidney uptake was slightly reduced from 1 h to 3 h post-injection. The tumor and kidney uptake of [^99m^Tc]Tc-EDDA-KL01127 and [^99m^Tc]Tc-EDDA-HTK03180 was greatly reduced with co-injection of the PSMA inhibitor 2-(phosphonomethyl)pentanedioic acid (2-PMPA, 0.5 mg), suggesting the uptake of [^99m^Tc]Tc-EDDA-KL01127 and [^99m^Tc]Tc-EDDA-HTK03180 in both tumors and kidneys is blockable and might be specific.

The biodistribution data of these PSMA-targeted tracers presented in Table 1 are consistent with the observations from their SPECT images. The tumor uptake values of [^99m^Tc]Tc-EDDA/HYNIC-iPSMA, [^99m^Tc]Tc-EDDA-HTK03180, [^99m^Tc]Tc-EDDA-KL01099, and [^99m^Tc]Tc-EDDA-KL01127 at 1 h post-injection were 10.3 ± 2.76, 18.8 ± 6.71, 5.36 ± 1.18, and 9.48 ± 3.42%ID/g, respectively, and their kidney uptake values were 45.3 ± 20.5, 91.8 ± 29.1, 65.9 ± 5.10, and 15.0 ± 14.7%ID/g, respectively. The spleen uptake at 1 h post-injection was higher for the tracers derived from the Lys-urea-Glu pharmacophore (23.4 ± 6.40%ID/g for [^99m^Tc]Tc-EDDA/HYNIC-iPSMA and 20.6 ± 7.77%ID/g for [^99m^Tc]Tc-EDDA-KL01099) than the tracers derived from the Lys-urea-Aad pharmacophore (3.14 ± 1.43%ID/g for [^99m^Tc]Tc-EDDA-HTK03180 and 0.23 ± 0.03%ID/g for [^99m^Tc]Tc-EDDA-KL01127). Similarly, the salivary gland uptake at 1 h post-injection was also higher for the tracers derived from the Lys-urea-Glu pharmacophore (7.77 ± 3.01%ID/g for [^99m^Tc]Tc-EDDA/HYNIC-iPSMA and 19.1 ± 4.05%ID/g for [^99m^Tc]Tc-EDDA-KL01099) than the tracers derived from the Lys-urea-Aad pharmacophore (2.35 ± 0.15%ID/g for [^99m^Tc]Tc-EDDA-HTK03180 and 0.25 ± 0.19%ID/g for [^99m^Tc]Tc-EDDA-KL01127). All tracers had minimal uptake (<2.2%ID/g) in the liver and intestines, indicating their excretions were mainly via the renal pathway. Among these tracers, [^99m^Tc]-EDDA-HYNIC-KL01127 had the highest tumor-to-background contrast ratios at 1 h post-injection (80.7 ± 42.7, 97.0 ± 42.9, 18.5 ± 3.54 and 0.87 ± 0.39 for tumor-to-bone, tumor-to-muscle, tumor-to-blood and tumor-to-kidney ratio, respectively). The tumor-to-background contrast ratios of [^99m^Tc]-EDDA-HYNIC-KL01127 further increased from 1 h to 3 h post-injection due to a relatively sustained tumor uptake (9.48 ± 3.42 to 7.58 ± 2.48%ID/g) but greatly reduced uptake in most of the background organs/tissues. Co-injection of [^99m^Tc]Tc-EDDA-HTK03180 with 2-PMPA (0.5 mg) significantly reduced its average uptake in the spleen, kidneys, LNCaP tumor xenograft, and salivary glands by 85, 95, 95, and 70%, respectively. Similarly, co-injection of [^99m^Tc]Tc-EDDA-KL01127 with 2-PMPA (0.5 mg) also significantly reduced its uptake in the spleen, kidneys, LNCaP tumor xenograft, and salivary glands by 43, 92, 95, and 32%, respectively. These data suggest that the uptake of [^99m^Tc]Tc-EDDA-HTK03180 and [^99m^Tc]Tc-EDDA-KL01127 in LNCaP tumor xenografts, spleen, kidneys, and salivary glands is blockable and might be specific.

### 2.4. In Vivo Stability

In vivo stability studies were conducted for all of the investigated tracers, and mouse plasma and urine samples (*n* = 3) were collected at 15 min post-injection for radio-HPLC analysis to check their intact fractions (Figures S5–S8). The intact fractions in urine and plasma samples were comparable for both [^99m^Tc]Tc-EDDA-HTK03180 (84.2 ± 5.9% vs. 82.2 ± 0.5%, Figure S6) and [^99m^Tc]Tc-EDDA-KL01099 (67.3 ± 26.7% vs. 72.9 ± 5.6%, Figure S7). However, a higher intact fraction in urine samples than in plasma samples was observed for both [^99m^Tc]Tc-EDDA/HYNIC-iPSMA (87.2 ± 3.1% vs. 14.9 ± 3.8%, Figure S5) and [^99m^Tc]Tc-EDDA-KL01127 (84.7 ± 8.5% vs. 32.1 ± 6.6%, Figure S8).

## 3. Discussion

High uptake of Lys-urea-Glu-derived PSMA-targeted radioligands in normal organs/tissues (such as kidneys, salivary glands, liver, and spleen) hinders the detection of PCa lesions [17] and could potentially cause toxicity when radiotherapeutic agents are used [18]. Accumulated evidence has suggested that the high uptake of Lys-urea-Glu-derived PSMA-targeted radioligands in kidneys and salivary glands might be due to binding to off-targets [19,20,21]. However, the identity of off-targets in kidneys and salivary glands remains controversial [22,23]. Recently we discovered that replacing Glu in the Lys-urea-Glu pharmacophore of [^68^Ga]Ga-PSMA-617 with a close analog Aad led to [^68^Ga]Ga-HTK03149 (Figure 1) with minimal uptake in kidneys and salivary glands [11]. Therefore, in this report, we investigated if the same phenomenon could be observed for the design of ^99m^Tc-labeled HYNIC-conjugated PSMA-targeted tracers.

In vitro competition binding assays showed that reduced average K_i_(PSMA) values were observed when replacing the Glu in the Lys-urea-Glu pharmacophore with Aad (3.11 nM for HYNIC-iPSMA vs. 8.96 nM for KL01027; 10.7 nM for KL01099 vs. 11.6 for HTK03180). This is consistent with our previous observation when comparing the Glu-containing Ga-HTK03041 (Figure 1, K_i_(PSMA)= 0.63 nM) with the Aad-containing HTK03149 (Figure 1, K_i_(PSMA)= 6.99 nM, Figure 1) [11,16]. These data support that compared with Aad, the Glu in the Lys-urea-Glu pharmacophore fits better into the glutamate pocket of the PSMA binding site. Previously, we noticed that tranexamic acid in the lipophilic linker is important for maintaining high PSMA binding affinity [15], and replacing the 2-naphthyalanine in Ga-PSMA-617 with 9-anthrylalanine also improved PSMA binding affinity [16]. However, replacing the 2-naphthyalanine in HYNIC-iPSMA with 9-anthrylalanine and the addition of a tranexamic acid between HYNIC and 9-anthrylalanine did not lead to better PSMA binding affinity (K_i_ = 3.11 nM for HYNIC-iPSMA and 10.7 nM for KL01099). Similarly, the same phenomenon was observed when comparing the K_i_ values of KL01127 and HTK03180 (8.96 vs. 11.6 nM). These data suggest that compared with HYNIC-tranexamic acid-9-anthrylalanine, HYNIC-2-naphthyalanine fits better into the S1 lipophilic accessory site of PSMA. In addition, the effect of modifying the lipophilic linker on PSMA binding affinity has to be considered based on the overall structure rather than the individual component of the lipophilic linker.

LogD_7.4_ measurements revealed that replacing the bicyclic 2-naphthyalanine with a tricyclic 9-anthrylalanine and the addition of a tranexamic acid increased the overall lipophilicity of the radioligands (LogD_7.4_ = −3.31 for [^99m^Tc]Tc-EDDA/HYNIC-iPSMA and −2.13 for [^99m^Tc]Tc-EDDA-KL01099; LogD_7.4_ = −3.37 for [^99m^Tc]Tc-EDDA-KL01127 and −2.56 for [^99m^Tc]Tc-EDDA-HTK03180). Despite higher lipophilicity, the logD_7.4_ values of [^99m^Tc]Tc-EDDA-KL01099 and [^99m^Tc]Tc-EDDA-HTK03180 are <−2.0; therefore, both tracers are still considered relatively hydrophilic, and they are expected to be excreted mainly via the renal pathway.

The SPECT images and biodistribution data of all the investigated tracers confirm the prediction from their low LogD_7.4_ values, as all of them were excreted mainly via the renal pathway. With relatively higher lipophilicity, [^99m^Tc]Tc-EDDA-KL01099 (LogD_7.4_ = −2.13) showed relatively higher average uptake in liver (2.19 vs. 0.23–0.55%ID/g), small intestine (1.57 vs. 0.26–0.53%ID/g), and large intestine (0.82 vs. 0.18–0.25%ID/g) than the other tracers at 1 h post-injection. The much lower uptake of Lys-urea-Aad-derived tracers ([^99m^Tc]Tc-EDDA-KL01127 and [^99m^Tc]Tc-EDDA-HTK03180) in spleen and salivary glands when compared with their corresponding Lys-urea-Glu derivatives ([^99m^Tc]Tc-EDDA-HYNIC/iPSMA and [^99m^Tc]Tc-EDDA-KL01099) is consistent with our previous findings when comparing the biodistribution data of [^68^Ga]Ga-HTK03041 [15] and [^68^Ga]Ga-HTK03149 [11]. This suggests that replacing Glu in the Lys-urea-Glu pharmacophore of PSMA-targeted tracers with Aad reduces their binding to the off-targets expressed in the spleen and salivary glands. However, the higher kidney uptake of the Lys-urea-Aad-derived [^99m^Tc]Tc-EDDA-HTK03180 (91.8%ID/g at 1 h post-injection) than the Lys-urea-Glu-derived [^99m^Tc]Tc-EDDA-KL01099 (65.9%ID/g at 1 h post-injection) was unexpected. Assuming the binding affinity to off-targets expressed in kidneys has been reduced with the replacement of Glu with Aad, the higher kidney uptake of [^99m^Tc]Tc-EDDA-HTK03180 than [^99m^Tc]Tc-EDDA-KL01099 could be due to factors other than off-target binding, which remain to be further investigated.

Despite comparable PSMA binding affinity (8.96–11.6 nM), the tumor uptake values of the reported three new tracers at 1 h post-injection vary from 5.36 ± 1.18%ID/g for [^99m^Tc]Tc-EDDA-KL01099 and 9.48 ± 3.42%ID/g for [^99m^Tc]Tc-EDDA-KL01127 to 18.8 ± 6.71%ID/g for [^99m^Tc]Tc-EDDA-HTK03180. This indicates that, besides PSMA binding affinity, other factors, such as binding to off-targets and pharmacokinetics, also contribute significantly to the overall tumor uptake of these tracers. This also explains why with an inferior PSMA binding affinity, [^99m^Tc]Tc-EDDA-KL01127 could have higher tumor uptake than [^99m^Tc]Tc-EDDA/HYNIC-iPSMA (10.3 ± 2.76%ID/g).

Among these tracers, [^99m^Tc]Tc-EDDA-KL01127 had only moderate uptake in LNCaP tumor xenografts. However, [^99m^Tc]Tc-EDDA-KL01127 also had the lowest background uptake, leading to higher tumor-to-background (bone, muscle, blood, and kidney) uptake ratios than those of other tracers, including the clinically validated [^99m^Tc]Tc-EDDA/HYNIC-iPSMA (Table 1) [9,24]. Therefore, [^99m^Tc]Tc-EDDA-KL01127 is promising for clinical translation for use to detect PCa with SPECT. The tumor-to-background uptake ratios of [^99m^Tc]Tc-EDDA-KL01127 further improved over time, and the tumor-to-kidney ratio reached 4.55 ± 1.45 at 3 h post-injection. Due to the 6 h half-life of ^99m^Tc, patients injected with [^99m^Tc]Tc-EDDA-KL01127 could potentially be imaged at a later time point to further enable the detection of PCa lesions adjacent to or in kidneys.

## 4. Materials and Methods

### 4.1. Synthesis of HYNIC-Conjugated PSMA-Targeted Ligands

HYNIC-conjugated PSMA-targeted ligands were constructed on Wang resin using a solid-phase peptide synthesis approach on the AAPPTec (Louisville, KY, USA) Endeavor 90 peptide synthesizer or CEM Corporation (Matthews, NC, USA) Liberty Blue automated microwave peptide synthesizer. *t*-Butyl-protected Lys(ivDde)-urea-Glu and *t*-Butyl protected Lys(ivDde)-urea-Aad pharmacophores were first constructed on Wang resin following previously reported procedures [11,16,25]. The ivDde protecting group was removed with 5% hydrazine in DMF. Subsequent addition of Fmoc-protected linker and Boc-protected HYNIC chelator utilized 5 equivalents of amino acid derivative, 5 equivalents of HATU, and 10 equivalents of DIPEA. Fmoc deprotection was conducted in between couplings with 20% piperidine in DMF. Ninhydrin tests were conducted to verify each coupling and deprotection step. Final resin cleavage and deprotection were conducted using 95% trifluoroacetic acid and 5% triisopropylsilane for 4 h. After filtration, the crude peptides were precipitated by the addition of diethyl ether, centrifuged, and purified by an Agilent HPLC system.

The Agilent (Santa Clara, CA, USA) HPLC system consisted of a model 1200 quaternary pump and a model 1200 UV absorbance detector (set at 220 nm). The operation of the HPLC system was controlled by Agilent ChemStation software, and the HPLC column used was a semi-preparative column (Luna C18, 5 µm particle size, 100 Å pore size, 250 × 10 mm) purchased from Phenomenex (Torrance, CA, USA). The eluate fractions containing the desired product were collected, pooled, and lyophilized using a Labconco (Kansas City, MO, USA) FreeZone 4.5 Plus freeze drier.

The identities of the PSMA-targeted ligands were verified by MS analysis using a Waters (Milford, MA, USA) Acquity QDa mass spectrometer with the equipped 2489 UV/Vis detector and e2695 Separations module. For HYNIC-iPSMA, the HPLC conditions were 20% acetonitrile and 0.1% formic acid in water, and the flow rate was 4.5 mL/min. Its retention time was 9.7 min, and the yield was 34%. ESI-MS: calculated [M+H]^+^ for C_31_H_37_N_7_O_9_ 652.3; found 652.3 (Figure S9). For HTK03180, the HPLC conditions were 27% acetonitrile and 0.1% formic acid in water, and the flow rate was 4.5 mL/min. Its retention time was 9.3 min, and the yield was 32%. ESI-MS: calculated [M+H]^+^ for C_44_H_54_N_8_O_10_ 855.4; found 855.6 (Figure S10). For KL01099, the HPLC conditions were 28% acetonitrile and 0.1% formic acid in water, and the flow rate was 4.5 mL/min. Its retention time was 6.7 min, and the yield was 18%. ESI-MS: calculated [M+H]^+^ for C_43_H_52_N_8_O_10_ 841.4; found 841.3 (Figure S11). For KL01127, the HPLC conditions were 20% acetonitrile and 0.1% formic acid in water, and the flow rate was 4.5 mL/min. Its retention time was 9.8 min, and the yield was 36%. ESI-MS: calculated [M+H]^+^ for C_40_H_52_N_8_O_10_ 666.3; found 666.2 (Figure S12).

### 4.2. Cell Culture

The LNCaP cells obtained from ATCC (via Cedarlane, Burlington, Canada) were cultured in RPMI 1640 medium supplemented with 10% FBS, penicillin (100 U/mL), and streptomycin (100 μg/mL) at 37 °C in a Panasonic Healthcare (Tokyo, Japan) MCO-19AIC humidified incubator containing 5% CO_2_. The cells were confirmed to be pathogen-free by the IMPACT Rodent Pathogen Test (IDEXX BioAnalytics, Columbia, MO, USA). Cells were grown until 80–90% confluence and washed with sterile phosphate-buffered saline (PBS, pH 7.4) and collected after 1 min trypsinization. The cell concentration was counted in triplicate using a hemocytometer and a manual laboratory counter.

### 4.3. In Vitro Competition Binding Assay

The in vitro competition binding assays were performed following our previously published procedures [15,26]. Briefly, LNCaP cells in RMPI medium with FBS, penicillin, and streptomycin were plated onto 24-well poly-D-lysine coated plates at 200,000 cells/well 48 h prior to binding assay. The growth medium was removed and the cells were rinsed with HEPES-buffered saline (50 mM HEPES, pH 7.5, 0.9% NaCl) two times before 400 µL of HEPES buffer were added to each well. Varying concentrations of PSMA-targeted ligands (50 µL) were added to each well in triplicate before adding 0.1 nmol/50 µL of [^18^F]DCFPyL. After the additions, plates were incubated and swirled at 37 °C. After 1 h incubation, the solutions were removed, and cells were washed with HEPES-buffered saline twice. Finally, 400 µL of 0.25% trypsin solution were added to each well. After 10 min incubation, cells were collected for counting using a PerkinElmer (Waltham, MA, USA) Wizard2 2480 automatic gamma counter. The K_i_ values were analyzed with GraphPad (San Diego, CA, USA) Prism 7 software using nonlinear regression fit.

### 4.4. Tc-99m Labeling

HYNIC-conjugated ligand (5 nmol) in ACN/H_2_O (according to previous HPLC purification conditions) was added to a mixture of EDDA (500 μg in 250 μL of 0.1 M NaOH), tricine (1 mg in 250 μL of 0.2 M PBS (pH = 6)), and SnCl_2_ (400 µg in 20 μL of 0.1 M HCl), followed by the addition of around 740 MBq of [^99m^Tc]NaTcO_4_. The reaction mixture was incubated at 80 °C for 20 min and then passed through a Waters (Milford, MA, USA) C18 Sep-Pak Plus Light cartridge (125 Å, 130 mg), which was pre-washed with ethanol (5 mL) and water (5 mL). The trapped Tc-99m labeled PSMA-targeted ligand was then eluted with ethanol (0.4 mL) and diluted with PBS to <10% ethanol content for further studies. Solid phase extraction via a C18 Sep-Pak cartridge was used to remove excess EDDA, tricine, SnCl_2_, and salts in buffers. Quality control was performed on an Agilent HPLC system consisting of a model 1200 quaternary pump, a model 1200 UV absorbance detector (set at 220 nm), and a Bioscan (Washington DC, USA) NaI scintillation detector. The operation of the HPLC system was controlled by Agilent ChemStation software, and the HPLC column used was an analytical column (Luna C18, 5 µm particle size, 100 Å pore size, 250 × 4.6 mm) purchased from Phenomenex. The HPLC conditions were 0–80% acetonitrile (over 20 min) and 0.1% formic acid in water, and the flow rate was 2.0 mL/min. The retention times for Tc-99m labeled HYNIC-iPSMA, HTK03180, KL01099, and KL01127 were 11.1, 12.9, 12.9, and 11.1 min, respectively.

### 4.5. LogD_7.4_ Measurement

LogD_7.4_ values were measured using the shake-flask method, as previously reported [15]. Briefly, an aliquot of Tc-99m labeled tracer (~1.85 MBq) was added to a vial containing 3 mL of *n*-octanol and 3 mL of phosphate buffer (0.1 M, pH 7.4). The mixture was vortexed for 2 min and then centrifuged at 3000 rpm for 15 min. A sample (0.1 mL) of the n-octanol and buffer layers was counted using the Perkin Elmer Wizard2 2480 automatic gamma counter. Values of LogD_7.4_ were calculated using the following equation: LogD_7.4_ = log_10_ [(counts in *n*-octanol phase)/(counts in buffer phase)].

### 4.6. Ex Vivo Biodistribution and SPECT/CT Imaging Studies

Ex vivo biodistribution and imaging studies were performed using male NOD-Rag1^null^IL2rg^null^ (NRG) mice according to the guidelines established by the Canadian Council on Animal Care and approved by the Animal Ethics Committee of the University of British Columbia. Mice were inoculated subcutaneously with 1.0 × 10^7^ LNCaP cells in the left shoulder while under anesthesia (2% isoflurane in oxygen). The mice were then used for biodistribution and imaging studies around 5–6 weeks after inoculation.

SPECT/CT imaging experiments were conducted using the MILabs (Houten, The Netherlands) U-SPECT-II/CT scanner. Approximately 20 MBq of tracer were injected into LNCaP tumor-bearing mice via a lateral caudal tail vein, and the mice were imaged at 1 h post-injection. For imaging, mice were sedated with 2% isoflurane in oxygen and positioned in the scanner on a heating pad with vitals monitored. For each image, a CT scan ran for 5 min to correct for localization and attenuation with voltage set at 60 kV and current at 615 µA followed by a 60 min static emission scan acquired in list mode using an extra ultra-high sensitivity multi-pinhole big mouse (2 mm pinhole size) collimator. Data were reconstructed using MILabs reconstruction software centered on the 140 keV photon peak. Reconstruction parameters used similarity-regulated ordered subset expectation maximization (32 subsets, 10 iterations) and a post-processing filter (Gaussian blurring) of 0.6 mm. Scatter correction was performed using the automatic triple energy window setting, and a calibration factor was applied, generating images in MBq/mL. Images were decay corrected to injection time and divided by the injected activity in PMOD (PMOD Technologies, ZH) to obtain quantitative images expressed as the percentage of the injected dose per gram of tissue (%ID/g). Data were then converted to DICOM for visualization using Inveon Research Workplace (Siemens Healthineers, Erlangen, Germany).

For ex vivo biodistribution studies, mice were injected with ~2 MBq of tracer via the tail vein. For blocking studies, mice were co-injected with 0.5 mg of 2-PMPA. At the pre-determined time points, mice were euthanized by CO_2_ asphyxiation. Blood and organs/tissues of interest were collected, weighted, and counted using the Perkin Elmer Wizard2 2480 automatic gamma counter.

### 4.7. In Vivo Stabilization

Male NRG mice (*n* = 3) were injected with ~10 MBq of Tc-99m labeled tracer and then euthanized at 15 min post-injection. Blood samples were collected via cardiac puncture, and serum proteins were precipitated with the addition of an equal volume of acetonitrile. The mixtures were centrifuged for 15 min to collect the clear plasma samples. Urine samples were also collected. Samples were filtered through a 0.45-micron filter and analyzed by HPLC using the same conditions for the quality control of Tc-99m labeled tracers.

### 4.8. Statistical Analysis

Statistical analyses were conducted using *t*-test or ANOVA using the Microsoft (Redmond, WA, USA) Excel software. A statistically significant difference was considered present when the adjusted *p*-value was less than 0.05.

## 5. Conclusions

Replacing Glu in the Lys-urea-Glu pharmacophore of [^99m^Tc]Tc-EDDA/HYNIC-iPSMA and [^99m^Tc]Tc-EDDA-KL01099 generated [^99m^Tc]Tc-EDDA-KL01127 and [^99m^Tc]Tc-EDDA-KL03180, respectively, with greatly reduce off-target uptake in spleen and salivary glands. Compared with the clinically validated [^99m^Tc]Tc-EDDA/HYNIC-iPSMA, its Lys-urea-Aad analog, [^99m^Tc]Tc-EDDA-KL01127, showed comparable tumor uptake but greatly reduced off-target uptake in most of normal organs/tissues including spleen, kidneys, and salivary glands, leading to superior tumor-to-background contrast ratios. [^99m^Tc]Tc-EDDA-KL01127 is, therefore, promising for clinical translation for SPECT imaging to detect PCa lesions, and the Lys-urea-Aad pharmacophore is also promising for the design of PSMA-targeted ligands, especially for radiotherapeutic application to minimize toxicity due to off-target uptake in normal organs/tissues.

## 6. Patents

Intellectual property rights related to compounds described in this manuscript have been licensed to Alpha-9 Theranostics, Inc.

## Figures and Tables

**Table 1:** Uptake of ^99m^Tc-labeled PSMA-targeted tracers in LNCaP-tumor bearing mice. The mice in the blocked groups were co-injected with 0.5 mg of 2-PMPA.

Organ Uptake (%ID/g)	[^99m^Tc]Tc-EDDA/HYNIC-iPSMA	[^99m^Tc]Tc-EDDA-HTK03180		[^99m^Tc]Tc-EDDA-KL01099	[^99m^Tc]Tc-EDDA-KL01127		
	1 h (*n* = 4)	1 h (*n* = 5)	1 h Blocked (*n* = 4)	1 h (*n* = 4)	1 h (*n* = 5)	3 h (*n* = 5)	1 h Blocked (*n* = 4)
Blood	0.64 ± 0.11	1.33 ± 0.25	1.98 ± 0.28	0.81 ± 0.14	0.54 ± 0.25	0.12 ± 0.03	0.59 ± 0.11
Small Intestine	0.42 ± 0.07	0.53 ± 0.09	0.55 ± 0.05	1.57 ±0.28	0.26 ± 0.07	0.10 ± 0.03	0.30 ± 0.03
Large Intestine	-	0.25 ± 0.01	0.23 ± 0.05	0.82 ± 0.22	0.18 ± 0.09	0.31 ± 0.10	0.13 ± 0.02
Spleen	23.4 ± 6.40	3.14 ± 1.43 ^†^	0.45 ± 0.10 ^†^	20.6 ± 7.77	0.23 ± 0.02	0.07 ± 0.01	0.13 ± 0.03
Liver	0.45 ± 0.07	0.55 ± 0.10	0.62 ± 0.06	2.19 ± 0.34	0.23 ± 0.09	0.14 ± 0.03	0.24 ± 0.04
Pancreas	1.29 ± 0.94	0.48 ± 0.13	0.31 ± 0.02	2.20 ± 0.28	0.13 ± 0.07	0.04 ± 0.01	0.11 ± 0.03
Stomach	0.13 ± 0.01	0.25 ± 0.10	0.20 ± 0.03	0.55 ± 0.16	0.22 ± 0.07	0.16 ± 0.03	0.25 ± 0.02
Kidneys	45.3 ± 20.5	91.8 ± 29.1 ^†^	4.75 ± 0.43 ^†^	65.9 ± 5.10	15.0 ± 14.7 ^†^	1.95 ± 1.41	1.17 ± 0.16 ^†^
Lungs	3.64 ± 1.10	1.85 ± 0.23	1.19 ± 0.12	4.42 ± 0.47	0.42 ± 0.18	0.11 ± 0.03	0.38 ± 0.08
Heart	0.64 ± 0.10	0.56 ± 0.11	0.56 ± 0.08	2.65 ± 0.42	0.13 ± 0.05	0.04 ± 0.01	0.17 ± 0.02
LNCaP Tumor	10.3 ± 2.76	18.8 ± 6.71 ^†^	2.11 ± 0.16 ^†^	5.36 ± 1.18	9.48 ± 3.42 ^†^	7.58 ± 2.48	0.45 ± 0.09 ^†^
Muscle	0.45 ± 0.13	0.28 ± 0.07	0.31 ± 0.12	0.98 ± 0.16	0.08 ± 0.05	0.03 ± 0.02	0.10 ± 0.03
Bone	0.47 ± 0.13	0.53 ± 0.08	0.39 ± 0.14	0.79 ± 0.30	0.13 ± 0.07	0.05 ± 0.02	0.14 ± 0.03
Brain	0.03 ± 0.01	0.05 ± 0.01	0.04 ± 0.00	0.04 ± 0.01	0.02 ± 0.01	0.01 ± 0.00	0.02 ± 0.00
Salivary Glands	7.77 ± 3.01	2.35 ± 0.15 ^†^	0.71 ± 0.34 ^†^	19.1 ± 4.05	0.25 ± 0.19	0.06 ± 0.01	0.17 ± 0.13
Tumor/Bone	22.3 ± 6.16	35.8 ± 12.4	5.96 ± 2.34	7.43 ± 2.48	80.7 ± 42.7	164 ± 48.3	3.29 ± 0.73
Tumor/Muscle	21.8 ± 0.87	71.1 ± 28.9	7.32 ± 2.07	5.70 ± 2.05	97.0 ± 42.9	286 ± 103	4.82 ± 1.52
Tumor/Blood	15.4 ± 2.13	14.5 ± 5.55	1.08 ± 0.21	6.88 ± 2.16	18.5 ± 3.54	61.2 ± 15.0	0.75 ± 0.04
Tumor/Kidney	0.33 ± 0.14	0.21 ± 0.07	0.45 ± 0.02	0.08 ± 0.02	0.87 ± 0.39	4.56 ± 1.45	0.39 ± 0.03

^†^ *p* < 0.05 between blocking and unblocked studies of candidates.

## Data Availability

The data generated from this study are available in the text and in the Supplementary Materials.

## Supplementary Materials

The following supporting information can be downloaded at: https://www.mdpi.com/article/10.3390/molecules28135120/s1, Figure S1: A representative QC radio-HPLC chromatogram of [^99m^Tc]Tc-EDDA/HYNIC-iPSMA; Figure S2: A representative QC radio-HPLC chromatogram of [^99m^Tc]Tc-EDDA-HTK03180; Figure S3: A representative QC radio-HPLC chromatogram of [^99m^Tc]Tc-EDDA-KL01099; Figure S4: A representative QC radio-HPLC chromatogram of [^99m^Tc]Tc-EDDA-KL01127; Figure S5: Representative radio-HPLC chromatograms of plasma and urine samples collected at 15 min post-injection from mice injected with [^99m^Tc]Tc-EDDA/HYNIC-iPSMA; Figure S6: Representative radio-HPLC chromatograms of plasma and urine samples collected at 15 min post-injection from mice injected with [^99m^Tc]Tc-EDDA-HTK03180; Figure S7: Representative radio-HPLC chromatograms of plasma and urine samples collected at 15 min post-injection from mice injected with [^99m^Tc]Tc-EDDA-KL01099; Figure S8: Representative radio-HPLC chromatograms of plasma and urine samples collected at 15 min post-injection from mice injected with [^99m^Tc]Tc-EDDA-KL01127; Figure S9: A representative MS spectrum of HYNIC-iPSMA; Figure S10: A representative MS spectrum of HTK03180; Figure S11: A representative MS spectrum of KL01099; Figure S12: A representative MS spectrum of KL01127.

## Sample Availability

Samples of the compounds are not available from the authors.
