# Supplementary material for: Synthesis and Evaluation of 99mTc-Labeled PSMA-Targeted Tracers Based on the Lys-Urea-Aad Pharmacophore for Detecting Prostate Cancer with Single Photon Emission Computed Tomography
Source: Molecules. 2023 Jun 29;28(13):5120. doi: 10.3390/molecules28135120 (PMC10343272; doi:10.3390/molecules28135120)
Supplement: Supplementary file 1 [file molecules-28-05120-s001.zip › molecules-2467043-supplementary.pdf]

SUPPLEMENTARY MATERIALS

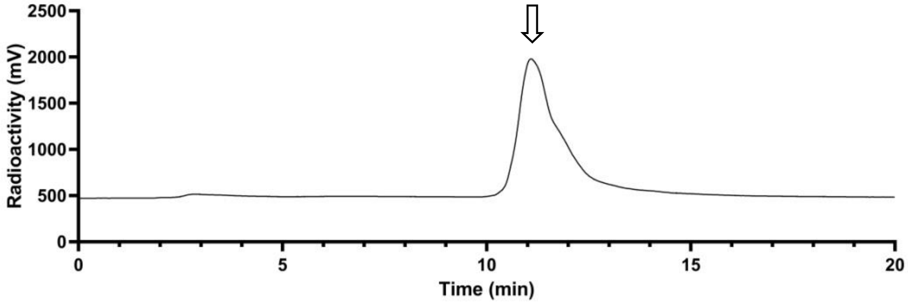

Figure S1. A representative QC radio-HPLC chromatogram of [^{99m}Tc]Tc-EDDA/HYNIC-iPSMA. The desired product peak is pointed by an arrow.

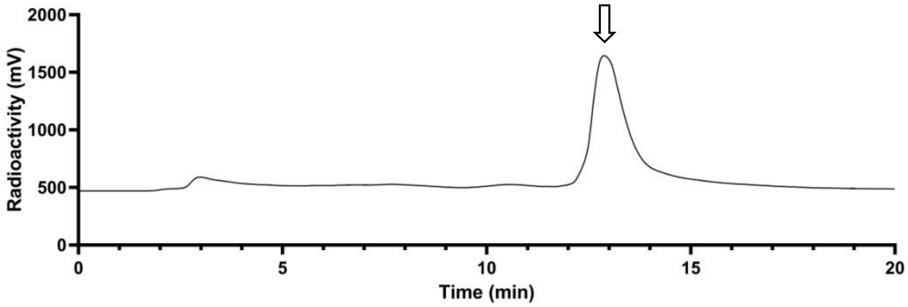

Figure S2. A representative QC radio-HPLC chromatogram of [^{99m}Tc]Tc-EDDA-HTK03180. The desired product peak is pointed by an arrow.

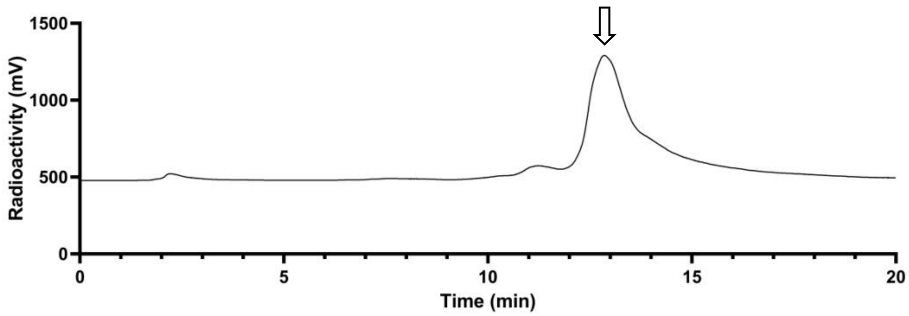

Figure S3. A representative QC radio-HPLC chromatogram of [^{99m}Tc]Tc-EDDA-KL01099. The desired product peak is pointed by an arrow.

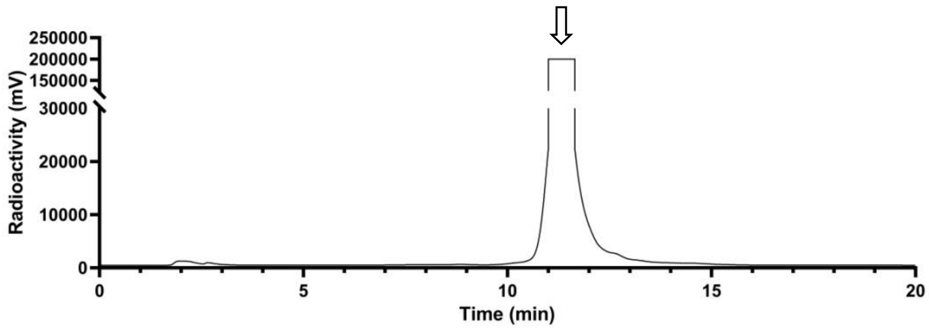

Figure S4. A representative QC radio-HPLC chromatogram of [^{99m}Tc]Tc-EDDA-KL01127. The desired product peak is pointed by an arrow.

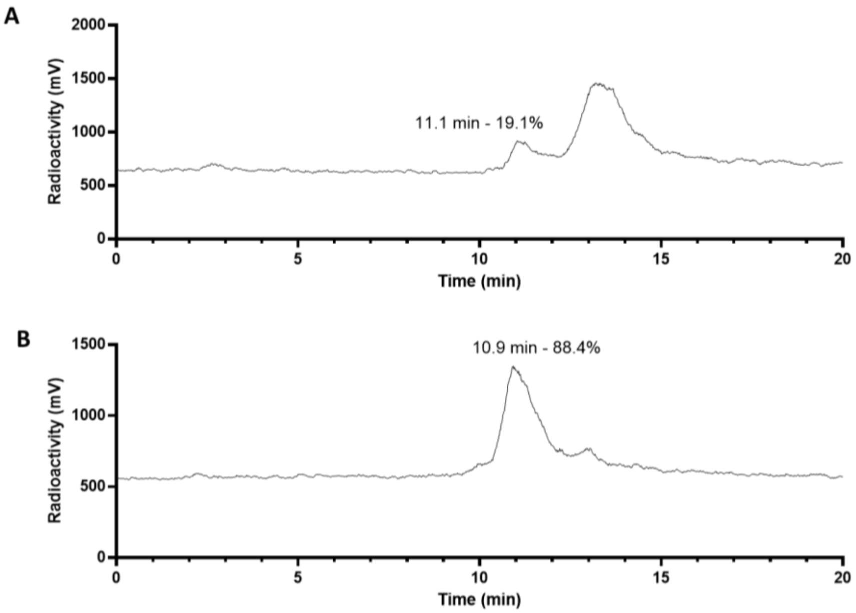

Figure S5. Representative radio-HPLC chromatograms of (A) plasma and (B) urine samples collected at 15 min post-injection from mice injected with [^{99m}Tc]Tc-EDDA/HYNIC-iPSMA. The peaks with the percentages are the intact tracer.

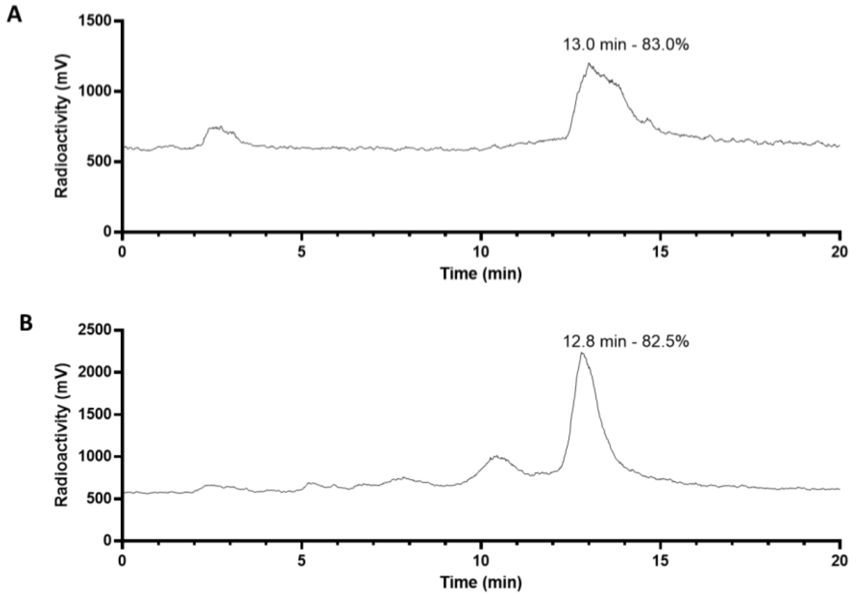

Figure S6. Representative radio-HPLC chromatograms of (A) plasma and (B) urine samples collected at 15 min post-injection from mice injected with [^{99m}Tc]Tc-EDDA-HTK03180. The peaks with the percentages are the intact tracer.

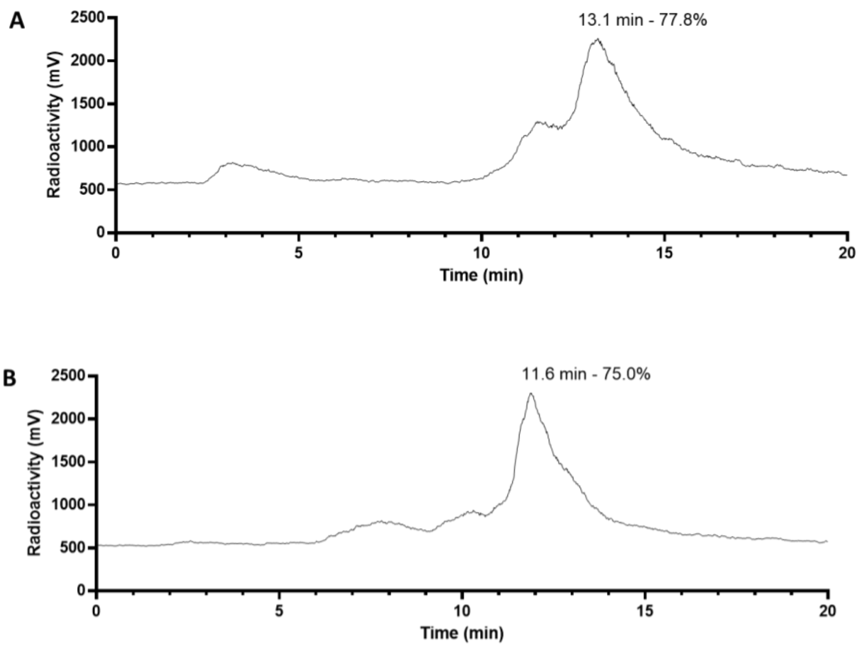

Figure S7. Representative radio-HPLC chromatograms of (A) plasma and (B) urine samples collected at 15 min post-injection from mice injected with [^{99m}Tc]Tc-EDDA-KL01099. The peaks with the percentages are the intact tracer.

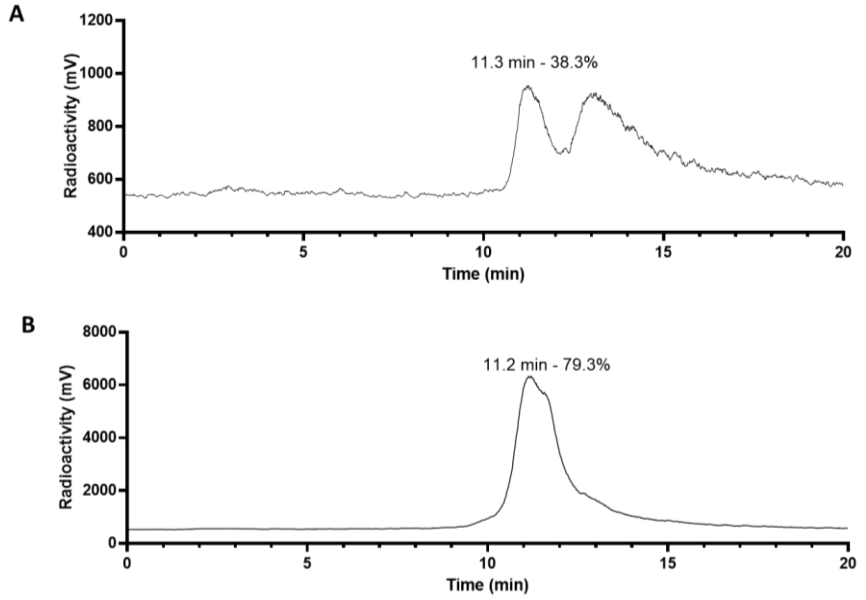

Figure S8. Representative radio-HPLC chromatograms of (A) plasma and (B) urine samples collected at 15 min post-injection from mice injected with [^{99m}Tc]Tc-EDDA-KL01127. The peaks with the percentages are the intact tracer.

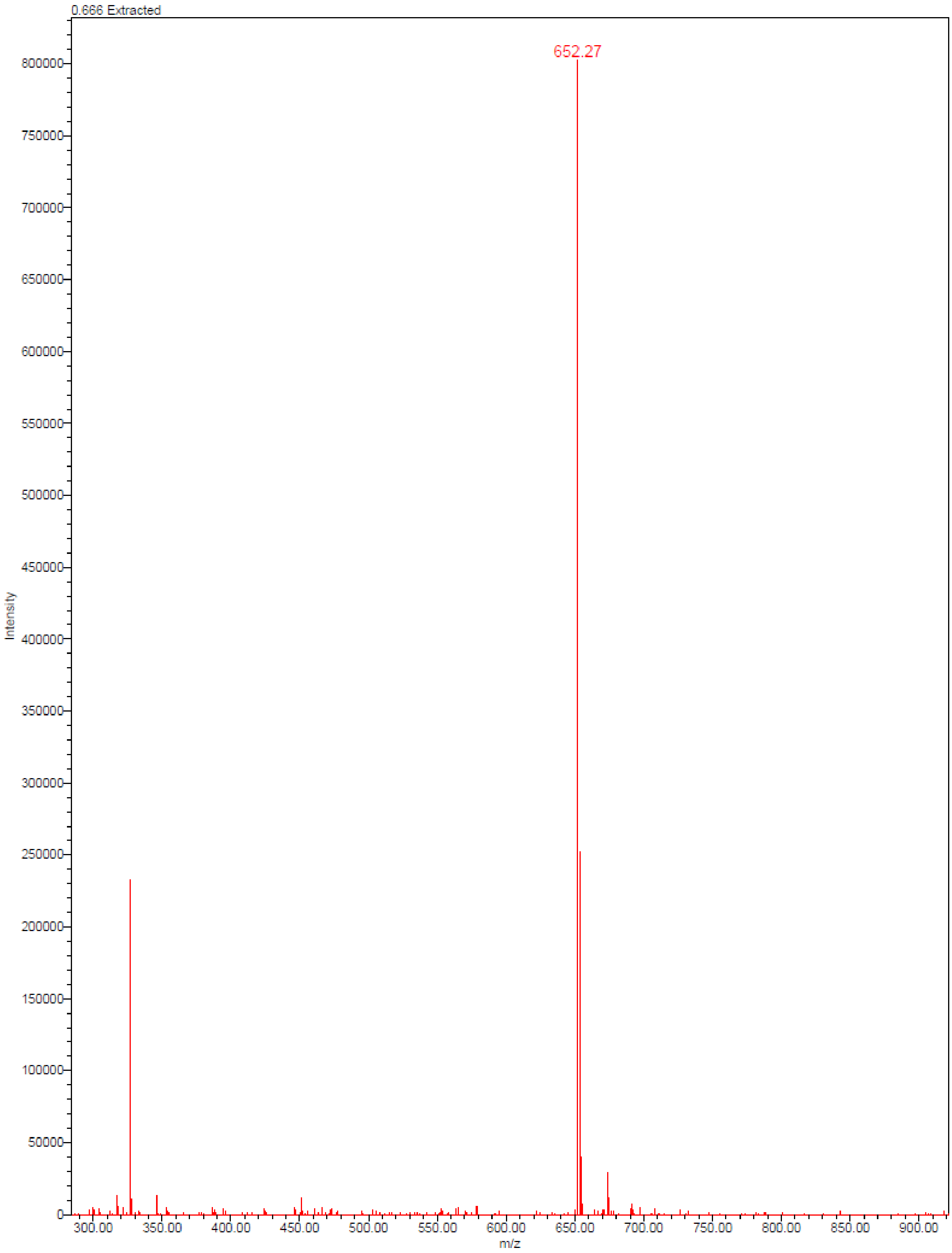

Figure S9. A representative MS spectrum of HYNIC-iPSMA. The calculated m/z value for $[M+H]^+$ is 652.3; observed 652.3.

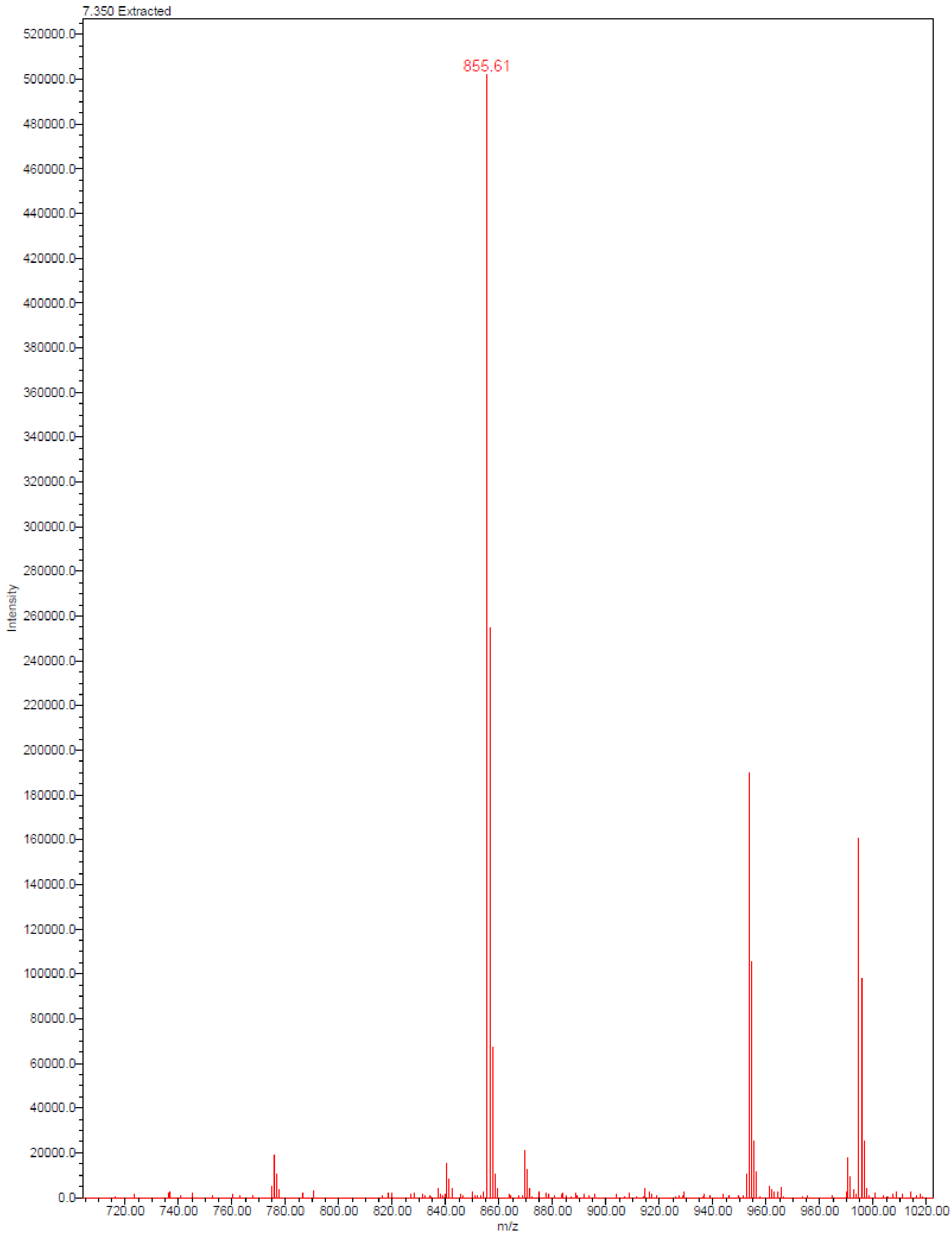

Figure S10. A representative MS spectrum of HTK03180. The calculated m/z value for $[M+H]^+$ is 855.4; observed 855.6.

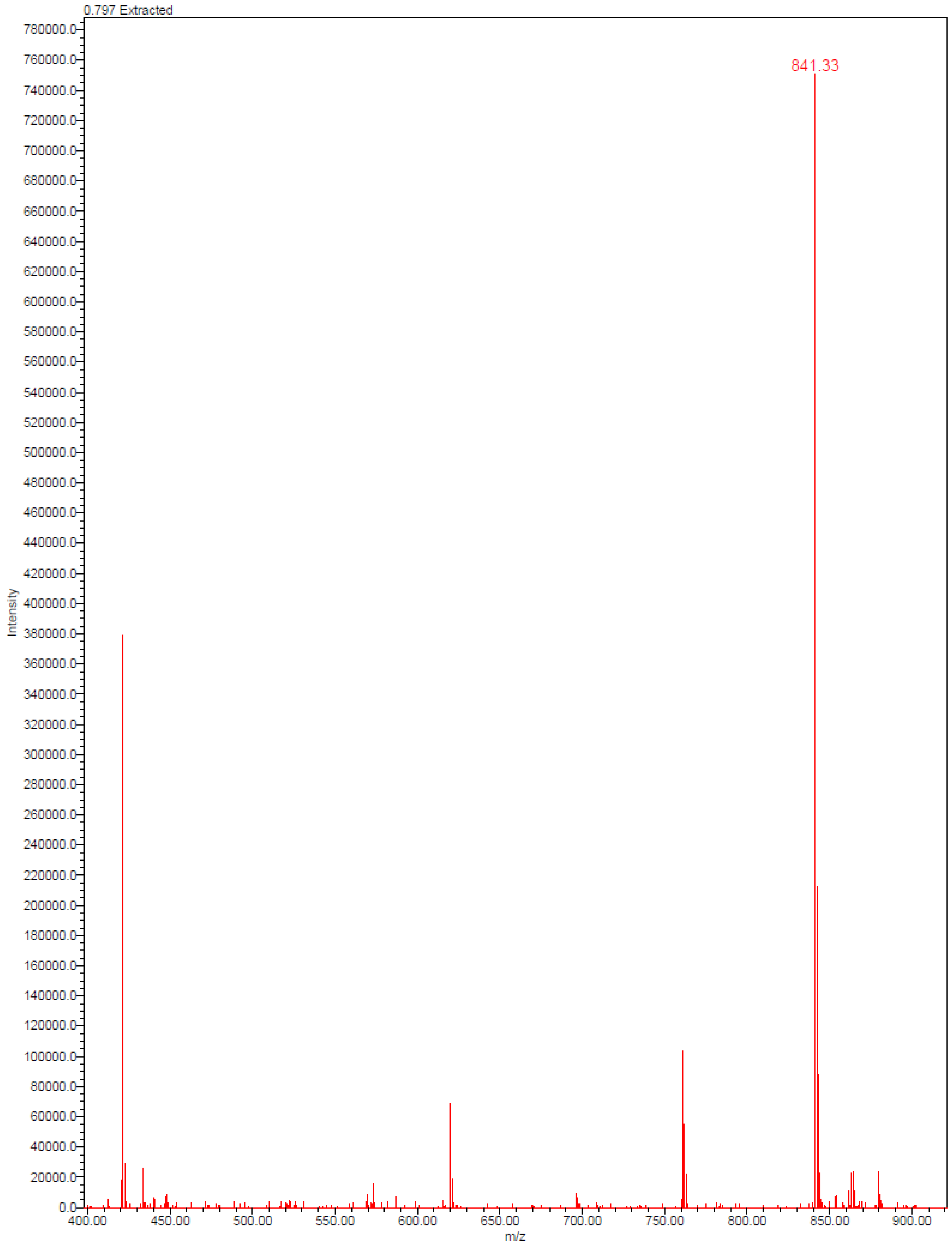

Figure S11. A representative MS spectrum of KL01099. The calculated m/z value for $[M+H]^+$ is 841.4; observed 841.3.

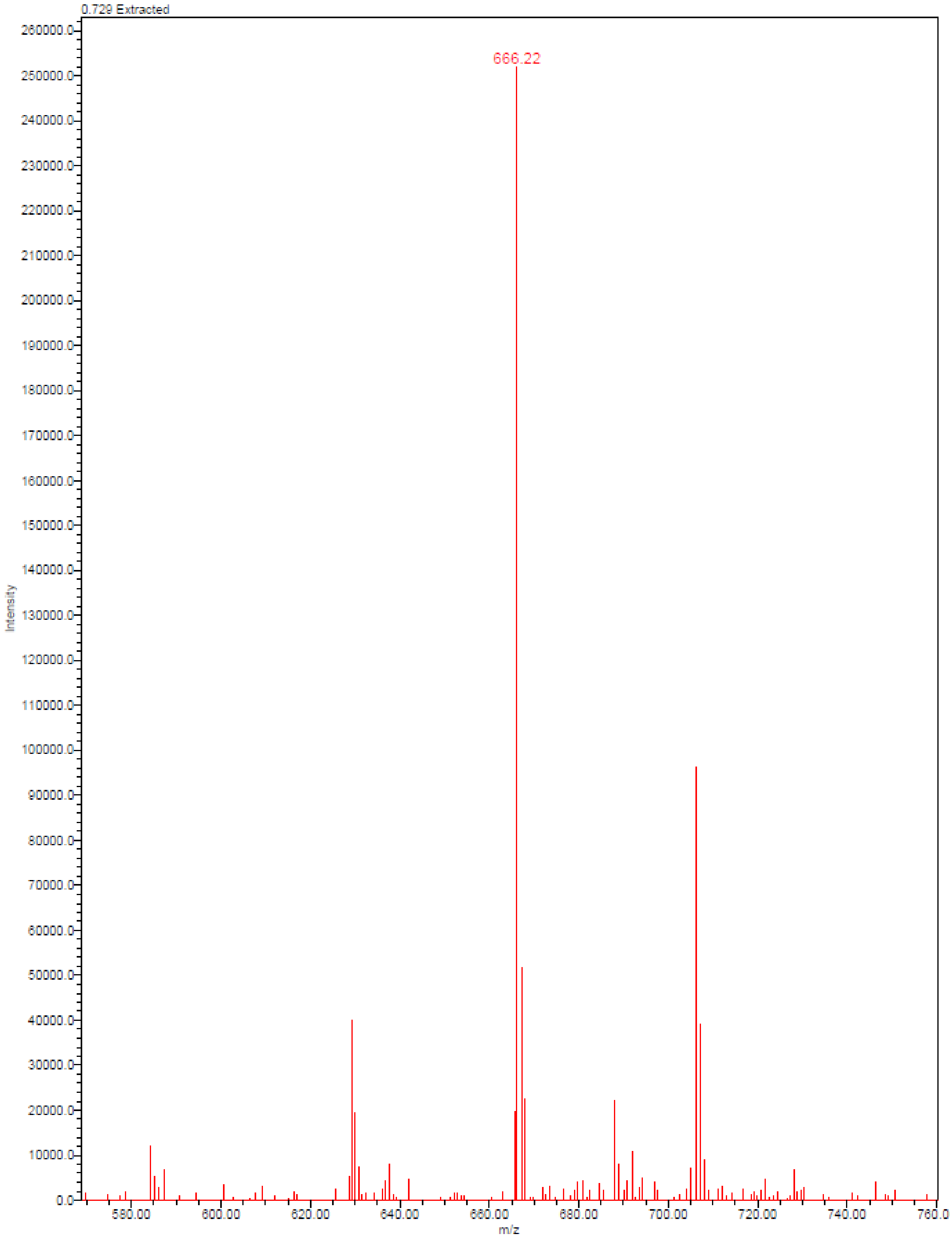

Figure S12. A representative MS spectrum of KL01127. The calculated m/z value for $[M+H]^+$ is 666.3; observed 666.2.
